# *In situ* full-field measurement of surface oxidation on Ni-based alloy using high temperature scanning probe microscopy

**DOI:** 10.1038/s41598-018-24656-w

**Published:** 2018-04-27

**Authors:** Yan Li, Xufei Fang, Zhe Qu, Siyuan Lu, Haicheng Li, Ting Zhu, Qingmin Yu, Xue Feng

**Affiliations:** 1 AML, Department of Engineering Mechanics, Tsinghua University, Beijing, 100084 China; 2 Center for Advanced Mechanics and Materials, Tsinghua University, Beijing, 100084 China; 3 Max-Planck-Institut für Eisenforschung, Max-Planck-Str. 1, 40237 Düsseldorf, Germany; 4 Woodruff School of Mechanical Engineering, Georgia Institute of Technology, Atlanta, GA 30332 USA; 5 School of Mechanics, Civil Engineering and Architecture, Northwestern Polytechnical University, Xi’an, 710072 China

## Abstract

We use *in situ* scanning probe microscopy (SPM) to investigate the high temperature oxidation of Ni-based single crystal alloys at the micro-/nanoscale. SiO_2_ micro-pillar arrays were pre-fabricated on the alloy surface as markers before the oxidation experiment. The SPM measurement of the oxidized surface in the vicinity of SiO_2_ micro-pillars was conducted real time at temperatures from 300 °C to 800 °C. The full-field evolution of oxide film thickness is quantitatively characterized by using the height of SiO_2_ micro-pillars as reference. The results reveal the non-uniform oxide growth featuring the nucleation and coalescence of oxide islands on the alloy surface. The outward diffusion of Ni and Co is responsible for the formation and coalescence of first-stage single-grain oxide islands. The second-stage of oxidation involves the formation and coalescence of poly-grain oxide islands.

## Introduction

Due to their excellent high temperature mechanical properties, corrosion resistance and high strength-to-weight ratio, Ni-based alloys have been widely used and hence extensively investigated over the past few decades^1–3^. The important related issues of Ni-based alloys include machinability, chemistry, microstructure, creep and oxidation behaviors, which have been studied using experimental, theoretical and computational methods^4–12^. In particular, the oxidation of Ni-based alloys has attracted considerable attention due to its common occurrence under extreme service conditions, such as high temperature environment, as well as due to an urgent need to improve the oxidation resistance of those alloys^10–12^. The oxide formation of Ni-based alloys is known to be greatly influenced by the nature of the starting surface, material purity and many other factors^13–16^. For instance, the correlation between surface growth and migration of substrate steps during the oxidadtion of NiAl(100) has been reported by Qin *et al*.^15^ and the mechanisms of oxidation rate of β-NiAl with embedded α-Al_2_O_3_ during polishing was investigated by Veal *et al*.^16^. Similarly, the oxide growth is also complicated and has been measured with different time laws, including for example the parabolic growth^17–19^, logarithmic rate law^20^, and a mixture of cubic and parabolic laws^21^.

Several conventional experimental methods have been used to study the oxidation of Ni-based alloys, including mass gain/loss method, oxygen uptake measurement, accelerated life test, etc^12,13,17–21^. These experimental methods have provided strong support for the investigations of oxidation kinetics, however, few of them have the ability of providing the important information of spatial variation of growing oxides, since the results are usually obtained by smearing out the regional difference of oxidation evolution. Recent studies via *in situ* high resolution transmission X-ray microscope^22,23^ and *in situ* transmission electron microscope^24,25^ have focused on the oxidation at the nano/atomic-scale and revealed a locally inhomogeneous oxidation behavior during the early stage of oxidation. However, these studies are limited to the incipient oxidation process without sufficient attention to oxide evolution as the oxide film is accumulated with time. Neither methods are able to quantitatively measure the full-field oxide growth rate of any arbitrary point on the oxide surface. Hence it is of great importance to develop an *in situ* method for studying the full-field evolution of the oxide layer. This is essential to understanding the oxidation mechanism and quantifying the oxidation process at the micro-/nanoscale.

Scanning probe microscopy (SPM) is an effective experimental tool for investigating the morphological evolution of materials from several 100 μm to sub-nanometer^26–29^. By properly choosing the scanning probe based on the specific material being investigated, SPM can be applied to study a broad range of materials such as metals and alloys, polymers and biomaterials for a variety of phenomena, such as growth and fabrication of crystals, thin films and nanoparticles, etc^30–35^. Moreover, due to its unique capability of mapping the real-time topography at the micro- to nano-scale at high temperatures, SPM has recently drawn increasing attention^31,36^. For example, high-temperature SPM has been used to study the temperature-dependent mechanical properties as well as the surface oxidation of materials^14,31,36^. Quantitative analysis of *in situ* SPM images provides new insights into the mechanisms underpinning the complicated oxidation processes.

**Figure 1:**
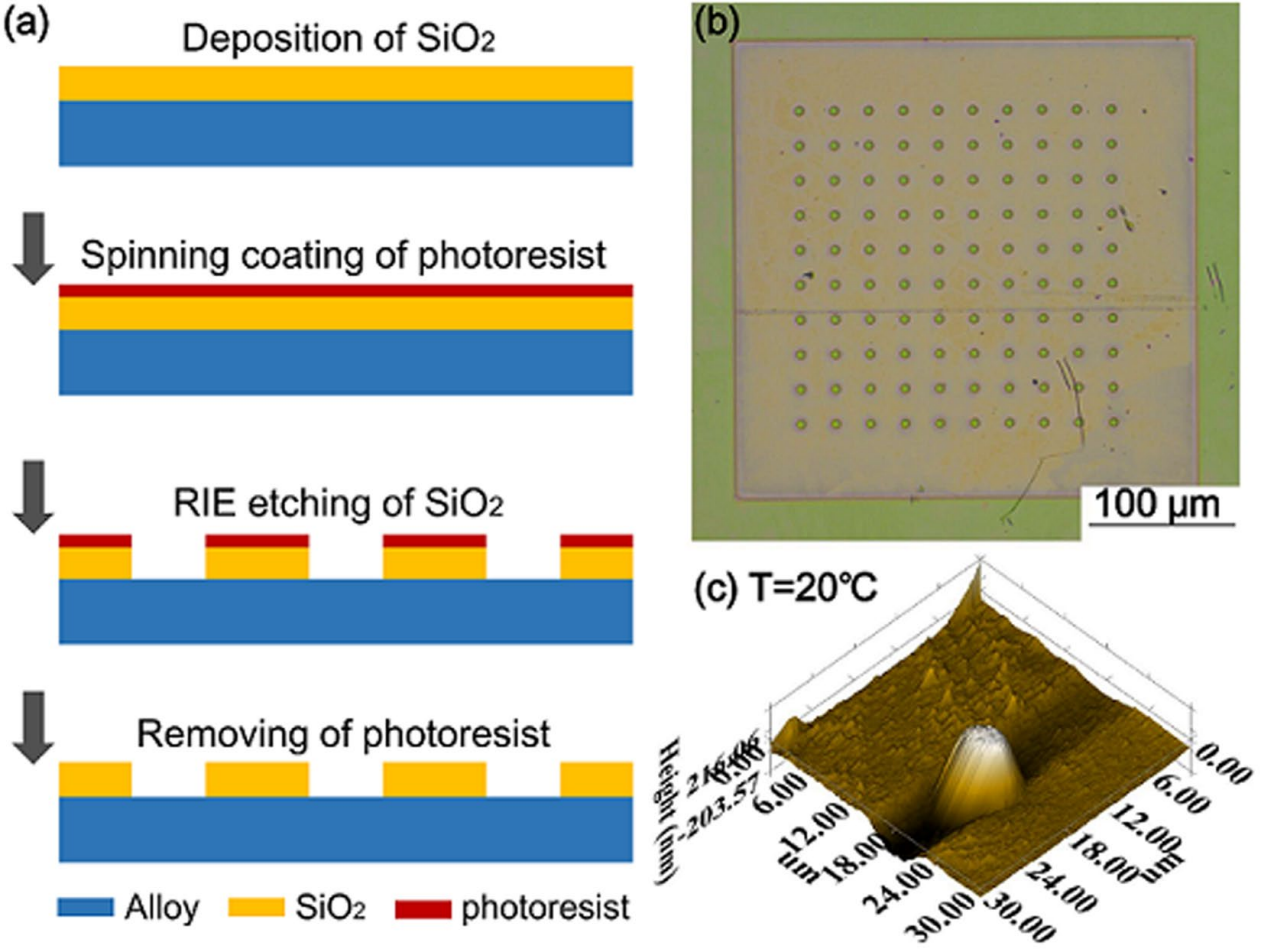
Preparation of an array of SiO_2_ micro-pillars on the surface of single crystal Ni-based alloy. (**a**) fabrication flow, (**b**) optical image of the pillar array; (**c**) SPM image of one SiO_2_ micro-pillar at room temperature.

In this work, *in situ* SPM was used to monitor the full-field profiles of oxides on the (111) surface of single crystal Ni-based alloy during oxidation at temperatures ranging from 300 °C to 800 °C. We fabricated an array of SiO_2_ micro-pillars as “markers” to facilitate the mapping of surface oxide evolution at the micro- to nano-scale. The process of fabricating the SiO_2_ micro-pillars on the surface of the Ni-based sample is shown in Fig. 1. More details are illustrated in Section Methods. A continuous increase of thickness of the oxide film in comparison to the non-oxidizing SiO_2_ micro-pillar was recorded with increasing temperature. This work bridges the gap of the oxidation studies at the atomic- and macro-scale. Our results provide new insights into the formation and growth of oxide islands on the alloy surface.

## Results and Discussion

### *In situ* measurement of full-field profiles of surface oxide

**Figure 2:**
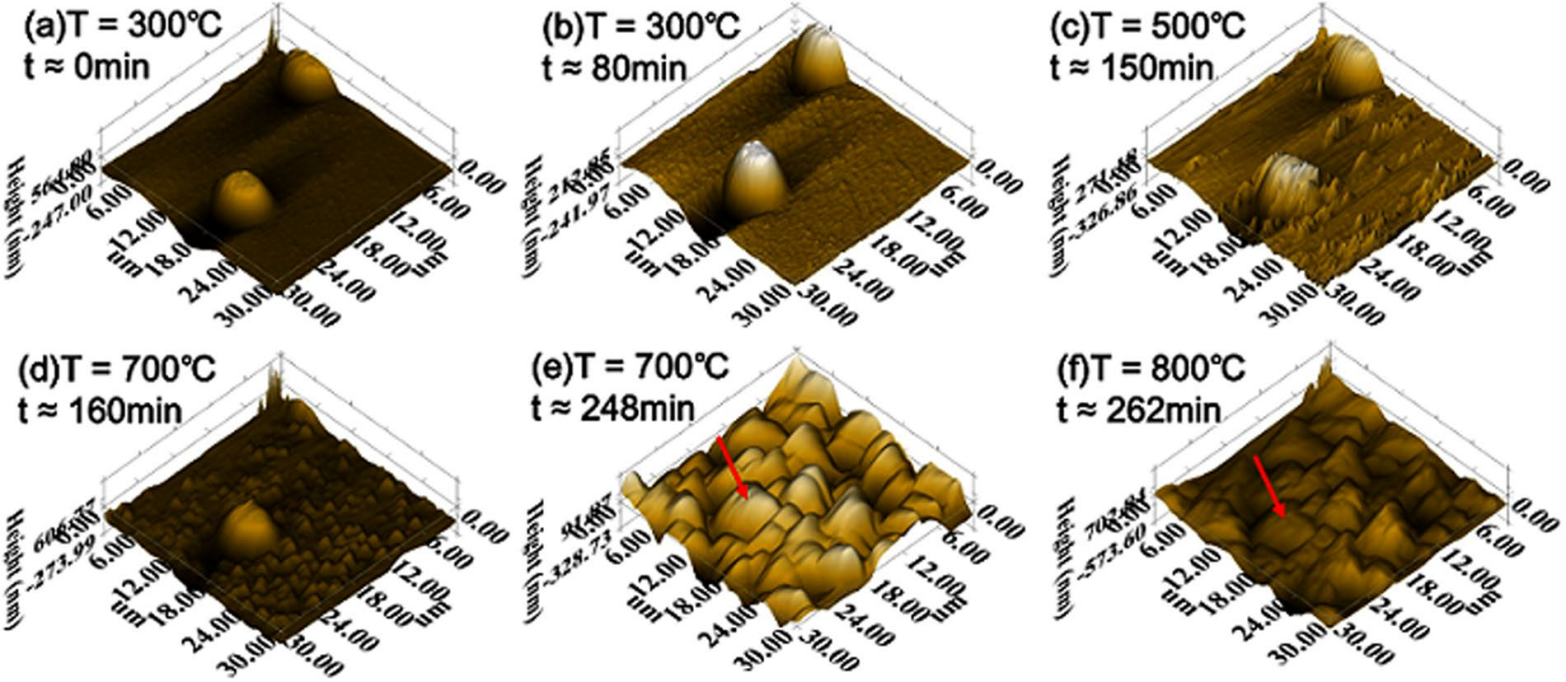
*In situ* SPM images showing the progressive surface oxidation of single crystal Ni-based alloy around the pre-fabricated SiO_2_ micro-pillar at different temperatures and oxidation times. For the ease of comparison among sub-figures, the micro-pillar is marked by a red arrow in (**e**) and (**f**).

During surface oxidation, measurement of the full-field profile of surface oxide was performed using SPM at a local area (30 × 30 μm^2^ in Fig. 2) around a SiO_2_ micro-pillar. Figure 2 shows the SPM images of oxidized surface obtained at different temperatures and times, with the SiO_2_ micro-pillar serving as a reference. When the oxidation experiment started at 300 °C, the SiO_2_ micro-pillar was clearly seen in Fig. 2(a). As the time increased to 80 min and the temperature remained at 300 °C, no obvious surface oxide was detected (Fig. 2(b)). This indicates the Ni-based alloy is stable around 300 °C. When the temperature reached 500 °C, small oxide islands emerged on the surface (Fig. 2(c)). The number of the islands increased as the temperature was raised to 700 °C (Fig. 2(d)), while the size of the islands remained similar to those at 500 °C. With a continuous increase of temperature, the islands grew bigger and began to merge with each other, such that the surface became considerably rough, as can be seen in Fig. 2(e) at 700 °C and Fig. 2(f) at 800 °C. Meanwhile, the micro-pillar became less distinguishable from the surrounding oxide with increasing temperature. As the temperature reached 700~800 °C in Fig. 2(e,f), the micro-pillar was almost indistinguishable from the surrounding oxide. Evidently, compared to the smooth surface at the beginning of oxidation experiment (Fig. 2(a)), the roughness of the sample surface progressively increased, especially at temperatures higher than 500 °C. This surface morphology of the oxide scale is apparently at variance with the classical theory of oxidation under an assumption of the uniformly thick oxide scale^37^.

We quantitatively characterized the full-field profile of the oxide scale around a SiO_2_ micro-pillar from the time series of SPM images. Specifically, the SiO_2_ micro-pillar was chosen as a reference marker, since it was highly resistant to oxidation and hence its absolute height remained unchanged in the temperature range of this experiment. On the other hand, the apparent height of the SiO_2_ micro-pillar decreased with respect to the oxidized surface during the growth of oxide scale. Hence, we set the top of the SiO_2_ micro-pillar as a reference plane and calculated the difference in height between any point on the oxide surface relative to the reference plane. In this way, the surface profiles of the oxide scale can be quantitatively mapped out in the course of oxidation.

**Figure 3:**
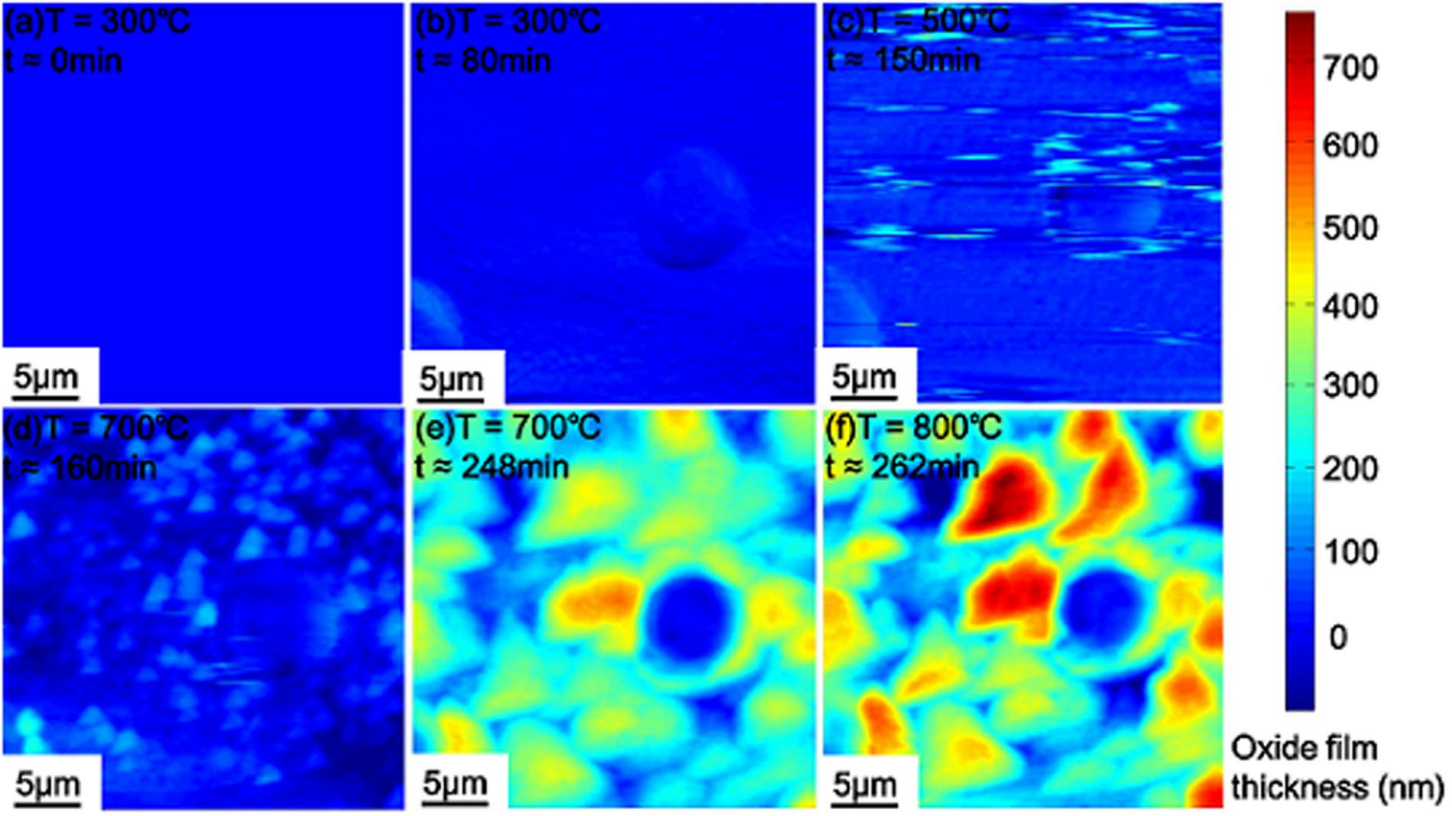
Full-field mapping of the oxide film thickness at different temperatures and times using the SiO_2_ micro-pillar as a reference marker. Each sub-figure corresponds to that in Fig. 2.

Figure 3 shows the calculated full-field of oxide film thickness, thus revealing a non-uniform oxidation process. Each map of oxide thickness in Fig. 3(a–f) was obtained based on the corresponding SPM image in Fig. 2(a–f). Notice that the big round blue spot in each sub-figure of Fig. 3 corresponds to the SiO_2_ micro-pillar. The uniform blue map in Fig. 3(a) and (b) corresponds to the case at *T* = 300 °C when no oxidation occurred. As seen from Fig. 3(c), tiny spots with light blue color appeared, indicating the thickness of the surface oxide film increases slightly as the temperature was raised from 300 °C to 500 °C. More island-shaped oxides appeared in Fig. 3(d–f) during oxidation at 700 °C and 800 °C. In particular, the red areas in Fig. 3(f) indicate the occurrence of local severe oxidation.

One advantage of using the SiO_2_ micro-pillar as a reference marker is that it facilitates the quantitative tracking of full-field of the oxide islands. For the oxide islands formed at *T = *700 °C and an earlier oxidation time of 160 min, their diameter and height are about 1 μm and 200 nm, respectively. As the oxidation time increases to 248 min at *T* = 700 °C, the oxide islands grew bigger and merged with each other, resulting in a decrease in the number of oxide islands (about 30 islands in Fig. 3(e)) but a concomitant increase in the height (about 500 nm) and diameter (about 5 μm) of the oxide islands. As shown in Fig. 3(e,f), the size of the oxide islands becomes close to that of the SiO_2_ micro-pillar, making it difficult to recognize the latter in the 3D SPM profiles in Fig. 2(e,f).

### Cross-section characterization using transmission electron microscope

**Figure 4:**
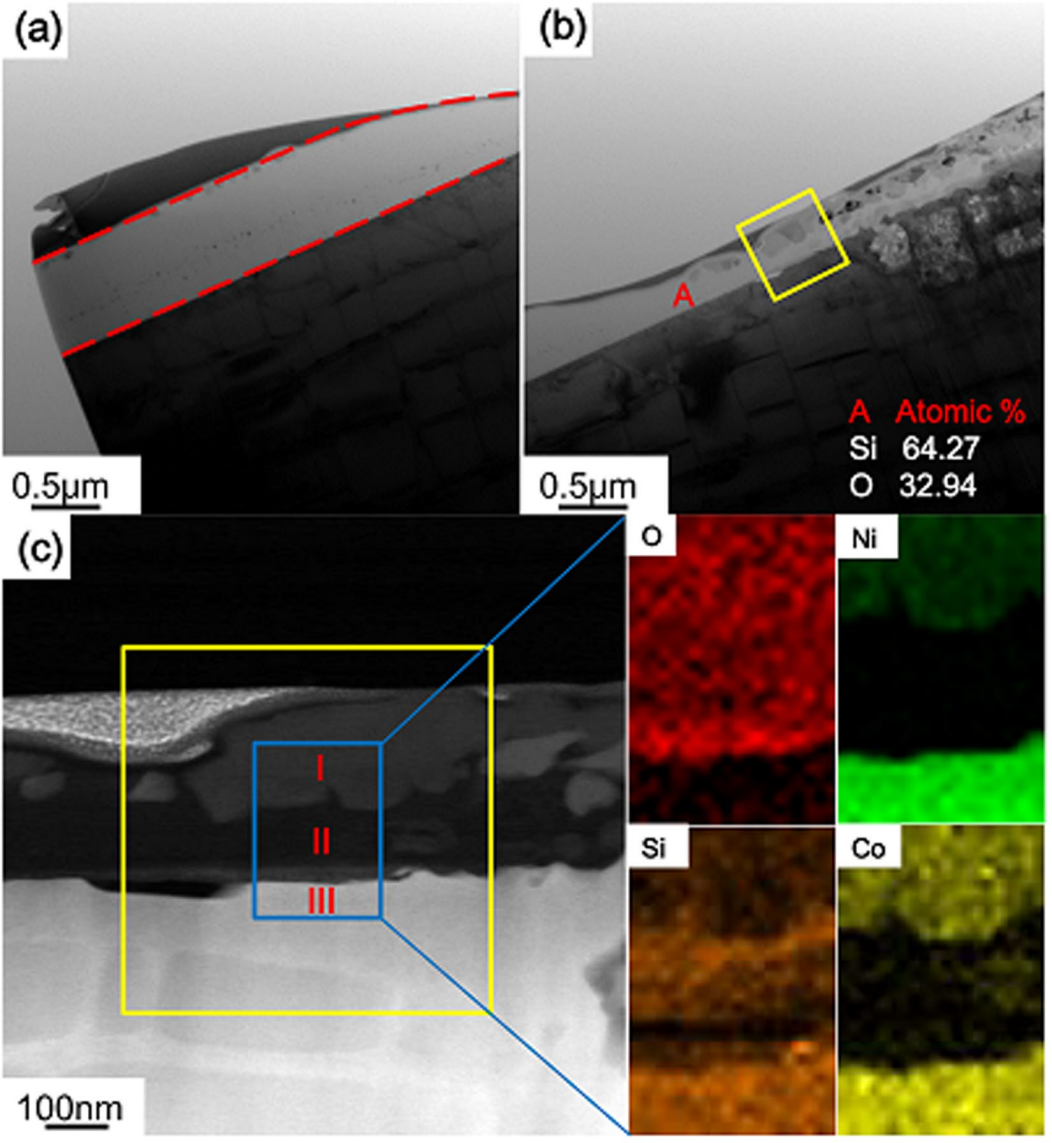
TEM images of (**a**) the SiO_2_ micro-pillar; (**b**) the oxide-substrate system; (**c**) enlarged view of the oxide-substrate system in (**b**) along with EDS elemental maps.

To gain further information on the formation process of the oxide islands, transmission electron microscope (TEM) was also used to investigate the cross-section of the oxide scale surrounding the SiO_2_ micro-pillar. For the ease of positioning, the material around the SiO_2_ micro-pillar shown in Figs 2 and 3 was chosen for TEM observation, as shown in Fig. 4(a–b). Energy Dispersive Spectrometer (EDS) elemental mapping was conducted to analyze the material compositions, as shown in Fig. 4(c).

The layer in between the two red dashed lines in Fig. 4(a) corresponds to the SiO_2_ micro-pillar. The representative oxide-substrate system is shown in Fig. 4(b). The region in the yellow square in Fig. 4(c) is the enlarged view of that in Fig. 4(b). A local area consisting of both the oxide film and alloy substrate was chosen for EDS elemental mapping. As shown in Fig. 4(c), the O element is distributed in the layers marked by I and II, with a sharp reduction in the substrate marked by III, while the distributions of Ni and Co are separated by a dark layer (II) due to depletion of Ni and Co elements. The concentration of Ni and Co elements in the outer oxide layer (I) is obviously smaller than that in the inner alloy substrate (III). Note that the Ni and Co depletion layer (II) is filled by Si and O, indicating that a SiO_2_ layer with a thickness of about 90 nm, overlapping with the depletion layer (II), exists on the sample surface before the oxidation occurs. The existence of such a thin layer of SiO_2_ is also confirmed by the quantitative element analysis at another location marked as A in Fig. 4(b). The thin layer of SiO_2_ is caused by the incomplete etching process. This thin layer of SiO_2_ may be responsible for impeding the outward diffusion of Cr or Al, since no sign of Cr_2_O_3_ or Al_2_O_3_ detected. It, however, unexpectedly facilitates to reveal the outward diffusion of Ni and Co elements^37^ during formation of an oxide scale on the outer layer of the sample surface, as discussed in the following section.

### Two-stage nucleation and coalescence of oxide islands

**Figure 5:**
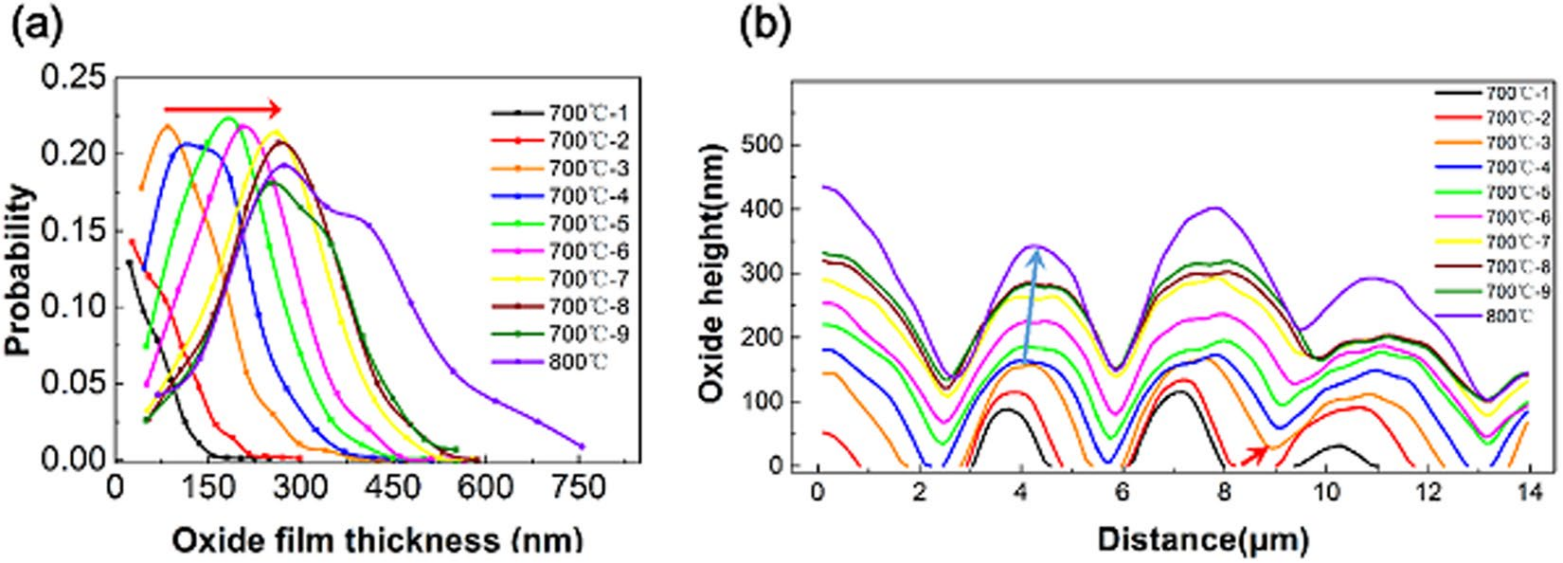
(**a**) Distribution probability of the non-uniform oxide film thickness and (**b**) cross-section view of the oxide islands as the oxidation evolves with time and temperature.

The 3D SPM images in Fig. 2 and the calculated maps of oxide film thickness in Fig. 3 clearly reveal the oxide islands formed on the sample surface, indicating a non-uniform growth of the oxide film. The TEM images in Fig. 4 reveal the outward diffusion of Ni and Co during the oxidation process. In order to better understand the oxidation process associated with outward diffusion of Ni and Co through the thin SiO_2_ layer, a statistical method is used to analyze the evolution of the non-uniform oxide film thickness at *T = *700 °C and 800 °C by using raw data from SPM scans. Specifically, we take 10% of the maximum oxide film thickness *h*_max_ in the respective SPM image as an increment for counting the number of data points falling into this thickness regime of the SPM profile. It follows that the statistical distribution (probability) of the non-uniform oxide film thickness could be calculated, as shown in Fig. 5(a).

Figure 5(a) reveals the oxide film thickness distributions for different times at the temperature *T* = 700 °C. The numbers of 1–9 indicate the sequence of SPM scanning. In addition, one curve obtained at *T* = 800 °C is added in Fig. 5(a) for comparison. It is seen from Fig. 5(a) that, as the oxidation process proceeds, the maximum oxide film thickness increases. For the first two curves (*T* = 700 °C-1 and 2), the thinnest oxide film accounts for the highest distribution probability while the thickest oxide film accounts for the lowest. For the third to seventh curves (*T* = 700 °C-3 to 7), the distribution of the oxide film thickness varies significantly, where the largest portion of the oxide film thickness falls in between the thinnest and the thickest oxide film. Moreover, the distribution peak (i.e. the highest probability) of the oxide film thickness shifts to a larger thickness. The shifting direction of the distribution peak is indicated by the red arrow. For the last two curves at 700 °C (*T* = 700 °C-8 and 9) and the curve at 800 °C, the oxide film thickness corresponding to the peak distribution remains almost unchanged, while the maximum distribution decreases gradually at 700 °C and increases slightly at 800 °C.

Figure 5(b) shows a more direct view of the evolving morphology of the oxide islands by extracting the cross-sectional profiles of the oxide islands. The oxide islands nucleate and grow at *T* = 700 °C-1 to 2. During this stage, the distribution of the oxide film thickness is monotonic (see Fig. 5(a)), meaning that the islands have not yet merged with each other (Fig. 5(b)). Thus, these two curves represent the process of nucleation of the oxide islands undergoing independent growth. As the oxidation proceeds, the oxide islands grow bigger and the neighboring oxide islands coalesce, as indicated by the red arrow in Fig. 5(b). Once the coalescence process continues (see the curve at *T* = 700 °C-3 to 8), the distribution of the oxide thickness is no longer monotonic in Fig. 5(a). Hence, the non-monotonic distribution of oxide film thickness in Fig. 5(a) reflects the coalescence process of the growing oxide islands, as indicted by the blue arrow in Fig. 5(b).

**Figure 6:**
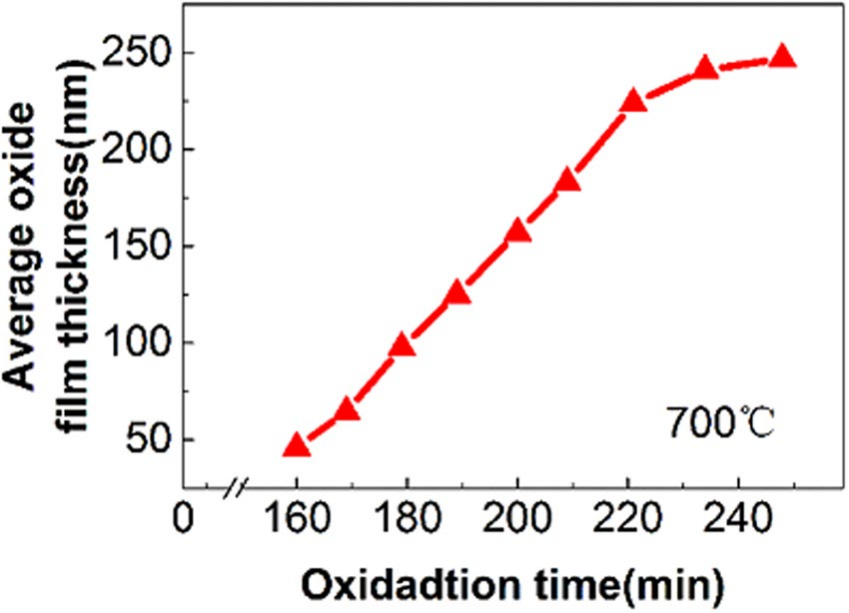
Average thickness of oxide film versus oxidation time at *T* = 700 °C.

Furthermore, we calculate the average oxide film thickness versus oxidation time using the conventional assumption of uniform growth of oxide scale. The result in Fig. 6 shows an initial linear increase of average film thickness with time, followed by a parabolic growth. The initial linear growth and subsequent parabolic growth of the oxide film are consistent with the previous reports by other researchers^17–20^. The initial linear law refers to reactions whose rate is controlled by a surface-reaction step, corresponding to the first-stage oxide island nucleation and growth. The followed parabolic law suggests a rate-determining process by the diffusion of Ni and Co through the scale, facilitating the second-stage oxide island growth. This result suggests that the some conventional method of evaluating the oxide growth may not accurately reflect the non-uniform formation and growth of oxide islands, particularly during the early stage of oxidation.

**Figure 7:**
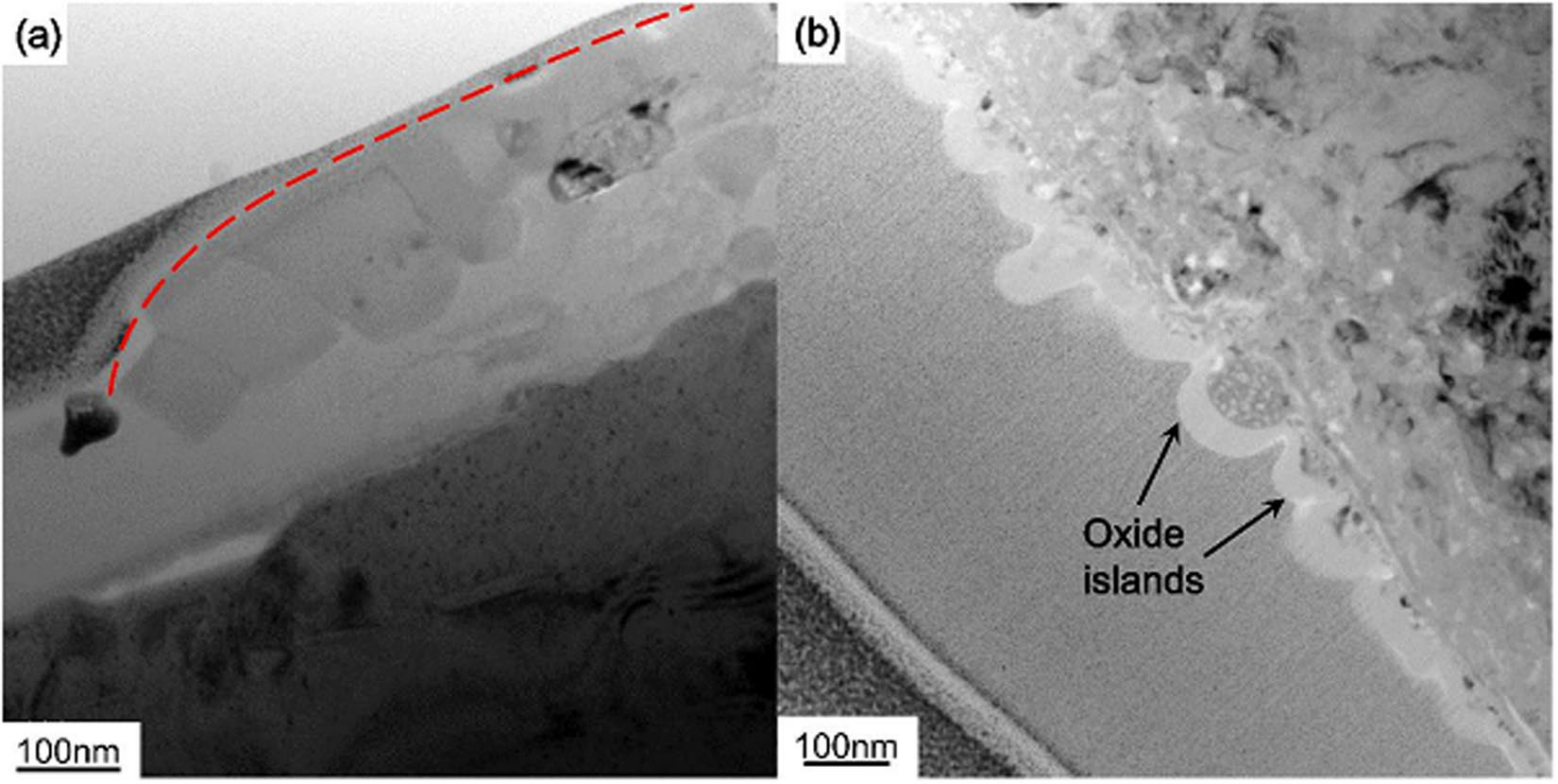
TEM images showing (**a**) an oxide island consisting of poly-grains and (**b**) small oxide islands after 30 min oxidation at *T* = 600 °C.

**Figure 8:**
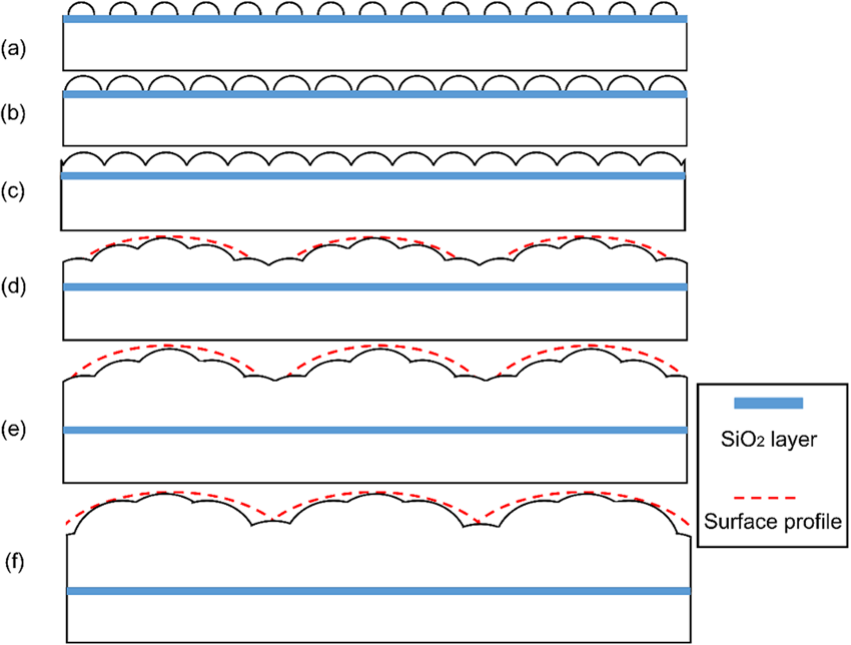
Schematic illustration of the two-stage oxidation. (**a**–**c**) The first stage of nucleation and coalescence of single grain oxide islands. (**d**–**f**) The second stage of nucleation and coalescence of poly-grain oxide islands.

By comparing the TEM images of oxide cross sections with the SPM maps of surface profiles, we find that the oxide islands in Figs 3 and 5 contain several to tens of small oxide grains. In Fig. 7(a), the red dashed line represents one large oxide island in the SPM map of Fig. 5(b). This island is composed of several small oxide grains. In contrast, Fig. 7(b) shows the TEM image from an independent oxidation experiment of a well-polished (110) single crystal of Ni-base alloy. It is seen that small oxide islands with diameters around 80 nm form on the surface after oxidization at *T* = 600 °C for 30 min. The combined results in Fig. 7(a,b) indicate that much smaller oxide islands form on the surface at a shorter time (30 min) and lower temperature (*T* = 600 °C). This is the first stage of oxidation involving the nucleation of the single grain oxide islands and their coalescence, which has been experimentally observed on the Cu surface during oxidation^38^. At longer oxidation time and higher temperature, as is the case in Figs 2–5, the second stage of nucleation and coalescence of poly-grain oxides occur on the surface. Similar phenomenon has been investigated during the oxidation process of 99.95 pct Ni^39^. The two stages of oxidation is schematically illustrated in Fig. 8.

To depict a more complete picture of oxidation in our experiment, we note that the oxidation process of single crystal Ni at the very early stage presumably involves the chemisorption, nucleation and lateral growth of extremely small oxide islands of approximately several nanometers in thickness, as discussed in detail in an early review article^40^. These small oxide islands continue to grow and coalesce, similar to Fig. 7(b). During this stage of oxidation, Ni and Co atoms diffuse through both the SiO_2_ layer and the oxide layer to react with the oxygen at the oxide-gas interface and form the oxide islands.

In order to further understand the mechanism for the occurrence of poly-grain oxide islands at the second stage, we compare the formation of the second-stage oxide islands with the surface roughening or undulation on the originally flat surface. A nominally flat but in reality slightly undulated surface at small scale is configurationally unstable during its growth or thickening^41–43^, which is attributed to the stress-induced surface instability^44^. During the second stage of oxide islands growth, the oxide islands are in conjunction with each other, the lateral growth results in compressive stress. Such compressive stress has been extensively reported in literature^45–48^ and could be responsible for the surface instabiltiy. A critical wavelength, ${\lambda }_{cr}=\frac{\pi {U}_{s}E}{{\sigma }_{m}^{2}}$, is proposed to estimate and characterize the evolution of surface configuration^41,47^. Here ${\lambda }_{cr}$ is the critical wavelength, ${U}_{s}$ is surface energy of the oxide thin film and can be treated as a material constant, *E* is the modulus, and ${\sigma }_{m}$ is the stress within the film. An intrinsic wavelength ${\lambda }_{in}$ is assigned to a given surface which is nominally flat but slightly undulated. The surface is stable during the growth of the thin film and does not become rougher if ${\lambda }_{in} < {\lambda }_{cr}$. When ${\lambda }_{in} > {\lambda }_{cr}$, the surface is unstable and roughens during the film growth^41,47^.

Assume that for a given pre-polished specimen, the value of ${\lambda }_{in}$ is a constant determined by the surface morphology, while the increasing value of compressive stress during the growth of oxide film reduces ${\lambda }_{cr}$ according to ${\lambda }_{cr}=\frac{\pi {U}_{s}E}{{\sigma }_{m}^{2}}$. When the critical condition of ${\lambda }_{in} > {\lambda }_{cr}$ is reached, further surface undulation is promoted, as observed in the present experiment as large secondary island formation and illustrated in Fig. 8. Here we take ${U}_{s}=1N/m$, $E=175\,GPa$ for the oxide film, the compressive stress was taken to be ${\sigma }_{ox}\approx -\,0.8\,GPa$^48^, it yields a critical wavelength of ${\lambda }_{cr}=\frac{\pi {U}_{s}E}{{\sigma }_{m}^{2}}=859\,nm$. The second-stage oxide island has a length scale of 1.5~2 μm, which is larger than the critical wavelength and agrees with the above argument.

## Conclusions

*In situ* SPM was used to investigate the oxidation process on the surface of single crystal of Ni-based alloy at high temperatures. An array of SiO_2_ micro-pillar was pre-fabricated on the sample surface as markers before the oxidation experiment. The surface profiles near the SiO_2_ micro-pillar was measured *in situ* in the temperature range of 300 °C to 800 °C. The *in situ* high temperature SPM enables the real-time quantification of the full-field oxide profile on material surfaces at the micro-/nanoscale, bridging the gap between the atomic-scale and macro-scale characterization of oxidation. The following conclusions can be drawn:

1. The novel method by combining SPM and non-reactive SiO_2_ pillar on the sample surface as reference marker yields quantitative results and can be applied for various materials systems besides Ni-base alloy.
2. The outward diffusion of Ni and Co through the SiO_2_ layer results in the nucleation and coalescence of single-grain oxide islands during the first stage of oxidation.
3. The second stage of oxidation involves the formation and coalesce of poly-grain oxide islands.

## Methods

### Material preparation

A single crystal Ni-based alloy (DD6, with 9.0 Co, 8.0 W, 5.7 Al, 4.3 Cr, 2 Mo, ≤ 0.20 Si, ≤ 0.1 Zr, ≤0.1 Cu, and Ni balanced in wt %) with the (111) surface is used in this work. The specimen underwent the following homogenizing, solution and aged heat treatments: 1290 °C for 1 hour + 1300 °C for 2 hours + 1315 °C for 4 hours, air cool + 1120 °C for 4 hours, air cooling + 870 °C for 32 hours, air cooling. The surface of the sample was first grinded using 400 to 2000-grit (with an interval of 200-grit) silicon carbide papers. Polishing was then performed with diamond paste with particle size of 0.5 μm. Vibration polishing using solutions containing nanosized particles was then carried out for 3 hours to remove the mechanical deformation layer and residual stress on the sample surface. The sample was cleaned using acetone and ethanol in ultrasonic bath before fabricating the SiO_2_ micro-pillar on the sample surface. The process of fabricating the SiO_2_ micro-pillars on the surface of the Ni-based sample is illustrated in Fig. 1. First, a thin layer (~600 nm in thickness) of SiO_2_ was sputter-deposited on the polished alloy surface. Then the photoresist (AZ5214E) was spin-coated onto the SiO_2_ layer, and cured at 120 °C for 2 minutes for solidification. Next we patterned the photoresist with pillar-shape by wet etching, which acts as mask for fabricating SiO_2_ micro-pillars. The SiO_2_ was etched into designed patterns by using Reactive Ion Etching (RIE). The residual photoresist was removed by RIE subsequently. Finally, an array of 10 × 10 SiO_2_ micro-pillars was prepared for the ease of fabrication. The diameter of the final micro-pillar was ~6.5 μm and the height was ~600 nm.

### Experimental procedure

*In situ* SPM was conducted by using a specific Berkovich indenter for the high temperature stage based on TI950 (Hysitron Inc., USA), a nanoindentation system with a displacement resolution of 0.02 nm and load resolution 1nN, respectively. A Berkovich diamond indenter with a radius 150 nm used as the SPM probe is brazed to a Macro-shaft with very low thermal conductivity for reducing the thermal conduction from the sample surface to the transducer. The heating stage integrated in the nanoindentation equipment is specially designed to reduce the thermal radiation in order to keep the constant temperature on the sample surface^11^. The heating stage consists of two heating plates, between which the sample is properly clamped and is subsequently heated to the pre-set temperature. Detailed description of this heating stage can be found elsewhere^11^. During high temperature oxidation, the SPM probe is first brought in contact with the sample with a very small contact load (2μN) and this contact is maintained until the temperature of the sample becomes thermally stable. This ensures a uniform distribution of temperature on the sample surface before a SPM scan is performed^28^.

We first carried out a SPM scan at room temperature to locate one of the SiO_2_ micro-pillar. The subsequent SPM scans were performed at the increased temperature of *T* = 300 °C, 500 °C, 700 °C and 800 °C, respectively. The rate of temperature increase was controlled by the equipment software, with an average rate of 3 °C/s. High-purify argon gas (Ar ≥ 99.9992%) was used for inert protection before the temperature reached 300 °C. This procedure was used to prevent the sample surface from being oxidized at temperature lower than *T* = 300 °C, so that the onset of the oxidation process and the oxide growth could be well controlled and monitored starting from *T* = 300 °C. Once the temperature reached *T* = 300 °C, the argon gas was stopped to allow the sample surface to be fully exposed in open air for oxidation. This allows us to accurately correlate both the starting time (i.e., *t* = 0 min) and temperature (*T* = 300 °C) corresponding to the onset of the oxidation process. When each target temperature (*T* = 300 °C, 500 °C, 700 °C and 800 °C) was reached with a fluctuation of ± 0.05 °C, several SPM scans were performed at each temperature to continuously record the surface evolution with a constant contact force of 2μN, as mentioned above. After the last SPM scan was finished at *T* = 800 °C, the indenter was withdrawn from the surface of the specimen, and heating was then turned off to allow the whole system to cool down.
